# The Crystal Water Affect in the Interaction between the Tenebrio Molitor Alpha-Amylase and Its Inhibitor

**DOI:** 10.1155/2008/469062

**Published:** 2008-05-11

**Authors:** Zhu Zhi-Fei, Ning Ting-Ting, Xu Zu-Min, Zhang Ge-Xin, Ma Yan-He

**Affiliations:** ^1^School of Chemical and Material Engineering, Jiangnan University, Wuxi, Jiangsu 214122, China; ^2^Institute of Microbiology, Chinese Academy of Sciences, Beijing 100080, China

## Abstract

Molecular dynamics simulation of the interaction between the Tenebrio molitor alpha-amylase and its inhibitor at different proportion of crystal water was carried out with OPLS force field by hyperchem 7.5 software. In the correlative study, the optimal temperature of wheat monomeric and dimeric protein inhibitors was from 273 K to 318 K. The the average temperature of experimentation is 289 K. (1) The optimal temperature of interaction between alpha-amylase and its inhibitors was 280 K without crystal water that was close to the results of experimentation. The forming of enzyme-water and inhibitor-water was easy, but incorporating third monomer was impossible. (2) Having analyzed the potential energy data, the optimal temperature of interaction energy between alpha-amylase and its inhibitors covering 9 : 1, 5 : 5, 4 : 6, and 1 : 9 proportion crystal water was 290 K. (3) We compared the correlative QSAR properties. The proportion of crystal water was close to the data of polarizability (12.4%) in the QSAR properties. The optimal temperature was 280 K. This result was close to 289 K. These findings have theoretical and practical implications.

## 1. INTRODUCTION

Alpha-amylases (1, 4-*α*-D-glucan-4-glucanohydrolase; EC3.2.1.1) are endoglycosidases which catalyze the hydrolysis of internal *α*-1, 4-D-glucosidic linkages in starch and dextrins, thereby generating smaller dextrins and oligosaccharides with a C_1_–OH group in the *α*-anomeric configuration [1].The enzyme is a subset of the alpha-amylases group of enzymes that are classified as glycosy1 hydrolase family 13 based on amino acid sequence similarity [2]. Alpha-amylases play a central role in carbohydrate metabolism of microorganisms, plants, and animals [3]. Furthermore, they are widely used in food and starch processing industry and, after proteases, have become the most used enzymes in modern biotechnology [4].

Enzyme inhibitors are important tools of nature for regulating the activity of enzymes in cases of emergency. Plant seeds are known to produce a variety of enzyme inhibitors that are thought to protect the seed against insects and microbial pathogens. Proteinase inhibitors are the best studied of this group [5]; expression of proteinase inhibitor genes in transgenic plants provides protection against pathogens [6]. Comparatively less is known about the inhibitors of alpha-amylase which might, on the other hand, be equally attractive candidates for conferring pest resistance to transgenic plants since many of them inhibit both proteinases and alpha-amylase. Plants have evolved defense strategies to counteract these effects through enzyme inhibitors impeding the action of insect gut digestive alpha-amylases and reducing the postprandial glucose peaks [7]. Expression of plants inhibitor genes in transgenic plants provides protection against pathogens (for a review, see Ryan). Comparatively less is known about the inhibitors of alpha-amylases which might, on the other hand, be equally attractive candidates for conferring pest resistance to transgenic plants since many of them inhibit both proteinases and alpha-amylases [8]. The inhibitor has potentials in various fields, from the treatment of diabetes to crop protection. Therefore, we are very interested in which mechanisms and optimal condition of the inhibitor exerted on alpha-amylases.

The major alpha-amylases inhibitor (AAI) present in the seeds of Amaranthus hypochondriacus, a variety of the Mex- ican crop plant amaranth, is a 32-residue-long polypeptide containing 6 cysteines, with 1–4, 2–5, 3–6 three disulfide bridges. It is the shortest alpha-amylases inhibitor described so far which has no known close homologs in the sequence data bases [8]. The structural properties of alpha-amylases have been elucidated by NMR [9]. People are very interested in which mechanism and optimal condition of inhibitor exerted on alpha-amylases.

Quantitative structure-activity relationship (QSAR) represents an attempt to relate structural descriptors of molecules with their physicochemical properties and biological activities. It is widely used for the prediction of physicochemical properties in chemical, environmental, and pharmaceutical areas [10, 11]. The main steps implicated in this method include data collection, molecular descriptor selection and procurement, correlation model development, and finally model evaluation. At present, many types of molecular descriptors have been proposed to describe the structural features of the molecules [12, 13]. The success of QSAR approach can be explained by the insight offered into the structural determination of chemical properties, and the possibility to estimate the properties of new chemical compounds without the need to synthesize and test them [14]. Recently, Anil Kumar has reported the development of useful QSAR models for antimicrobial activity [15–17] and anti-inflammatory activity [18].

In this work, we selected the structure of alpha-amylases from Tenebrio molitor larvae (containing 471 amino acid residues) and inhibitor from the amaranth. A number of crystal waters were distributed to alpha-amylases and their inhibitors manually using hyperchem 7.5 software according to different proportion via molecular dynamics simulation. The Tenebrio molitor alpha-amylase and the amaranth alpha-amylase inhibitor QSAR properties were calculated. The results showed that the crystal water had affected in the interaction between the alpha-amylases and their inhibitors. These findings have theoretical and practical implications.

## 2. MATERIALS AND METHODS

The structure of Tenebrio molitor alpha-amylase, the amaranth alpha-amylase inhibitor, and crystal water (con- taining 273 molecules) was taken from 1clv (http://www.rcsb.org/pdb/). (1) Alpha-amylase and inhibitor QSAR properties were calculated out to find out the correlation using OPLS force field by hyperchem 7.5 software. There are partial charges, surface area [approx.], surface area [grid], volume, hydration energy, log P, refractivity, polarizability, and mass of structural variance. (2) The Tenebrio molitor alpha-amylase, the amaranth alpha-amylase inhibitor, and crystal water were 3 monomers. 3 monomers formed 4 united molecules (enzyme-inhibitor, enzyme-water, inhibitor- water, and enzyme-inhibitor-water). (3) The partial crystal water formed united molecules with alpha-amylase or inhibitor. A number of crystal waters were distributed to alpha-amylase and its inhibitor manually according to different proportion (*E* : *I* = 9 : 1,8 : 2,…, 1 : 9). The energy of alpha-amylase, inhibitor, crystal water, and the united molecular structure was calculated out using OPLS force field by hyperchem 7.5 software. Calculated detail was the following text.

All modeling procedures, including energy minimization and molecular dynamics, were performed using the hyperchem 7.5 software. Energy calculations were carried out using the OPLS force field. Optimized molecular structure until the maximum energy derivative was lower than 0.1 kcal/moL (0.418 kJ/moL) in order to obtain a correct geometry. Dynamics simulation was performed using a time step of 0.5femtosecond, and the temperature was altering 10 K from 270 K to 370 K. There were 3 processes in simulation. Firstly, heating, from 0 K to simulation temperature using 7 K per step, heating time was 0.1 ps. Secondly, simulating, simulation time was 20 picoseconds in simulation temperature. Finally, annealing, from simulation temperature to 0 K using 7 K per step, annealing time was 0.1 picosecond [19].The system was kept for 20.2 picoseconds at each temperature. After simulation, we collected data of EPOT [20].

Recent research showed that enzymes had been used on all conformation not only during catalysis but also before catalysis. Since the protein motions necessary for catalysis were an intrinsic property of the enzyme, motion was localized not only to the active site but also to a wider dynamic network [21]. Thus, it can be seen that molecular state was taken on all possible conformation during reaction or else process. Therefore, in order to reflect energy during simulation, we carried out abnegating half potential energy data of starting simulation and averaging spare potential energy data (twenty thousand states between 10.1 picoseconds and 20.1 picoseconds). Gained data were regarded as potential energy at this temperature. We kept enzyme having enough number of state during the process of simulate temperature and avoided effective influence that system had been arrived at simulate temperature but was not likely to reach balance at the same time.

The energy of interaction was calculated from experimental data using the following equation [22–24]:

(1) $$\Delta E=E-(E_{1}+E_{2}).$$

Here, *E* was the overall energy of the binding system; *E*_1_ was the energy of alpha-amylase and crystal water; *E*_2_ was the energy of inhibitor and crystal water; Δ*E* was the interaction energy.

## 3. RESULTS AND DISCUSSION

### 3.1. The QSAR properties

Since predictions from any QSAR models cannot be intrinsically better than the experimental data employed to develop the model, the quality of the input data will greatly influence the QSAR model performance. In order to build a QSAR model with good generalized performance, a preliminary analysis for the quality of the data set (mainly the detection of outliers) was performed by modeling the complete set of alpha-amylase and its inhibitor.

The QSAR properties of alpha-amylase and its inhibitor were provided in Table 1.

Because the inhibitor and alpha-amylase were neutral molecules, so their partial charges were zero in Table 1. The proportion of surface area [approx.], hydration energy of inhibitor, and alpha-amylase was very small and neglected. But the proportion of surface area [grid], volume, log P, refractivity, polarizability, and mass were 16.1%, 15.8%, 0.1%, 13.0%, 12.4%, 7.2%, respectively. Therefore, the different distributed proportion of crystal water was possible close to these QSAR properties proportion.

### 3.2. Simulate optimal temperature among 3 monomers

**Figure 1:**
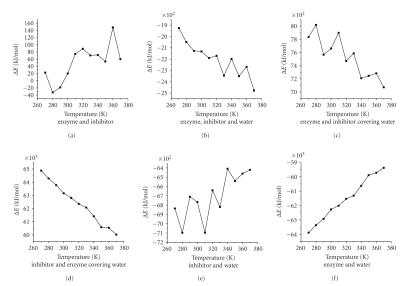
The respective chart of Δ*E* among 3 monomers.

We calculated the interaction energy among 3 monomers according to (1). The relation of the interaction energy with temperature was presented in Figure 1 (the respective chart of Δ*E* among 3 monomers). From Figure 1, the interaction energy between alpha-amylase and its inhibitor was negative at 280 K and 290 K, which showed that it was combined and reacted between alpha-amylase and its inhibitor. However, it was not combined between alpha-amylase and its inhibitor at the others temperature. The interaction energy was on the nadir at 280 K which was the optimal temperature between alpha-amylase and its inhibitor.

This information showed that the interaction energy between crystal water and alpha-amylase inhibitor was negative from 270 K to 370 K, which showed that they were combined and reacted among crystal water, alpha-amylase, and its inhibitor. As temperature increases, the interaction energy between alpha-amylase and its inhibitor tends to get smaller. However, the interaction energy between crystal water and alpha-amylase inhibitor was negative, and the numerical value was very big from 270 K to 370 K.The results showed that it was easy to combine between crystal water and alpha-amylase inhibitor.

Analysis of the results indicated that the interaction energy between alpha-amylase and its inhibitor was negative from 270 K to 370 K. The forming of inhibitor-water was easy, but the incorporating alpha-amylase was impossible.

The interaction energy between alpha-amylase and its inhibitor was negative, and the numerical value was very big from 270 K to 370 K. The forming of enzyme-water was easy, but the incorporating inhibitor was impossible.

The result was as follows: the forming of enzyme-water and inhibitor-water was easy, but the incorporating third monomer was impossible.

### 3.3. Simulate optimal temperature at the different distributed proportion of crystal water

**Figure 2:**
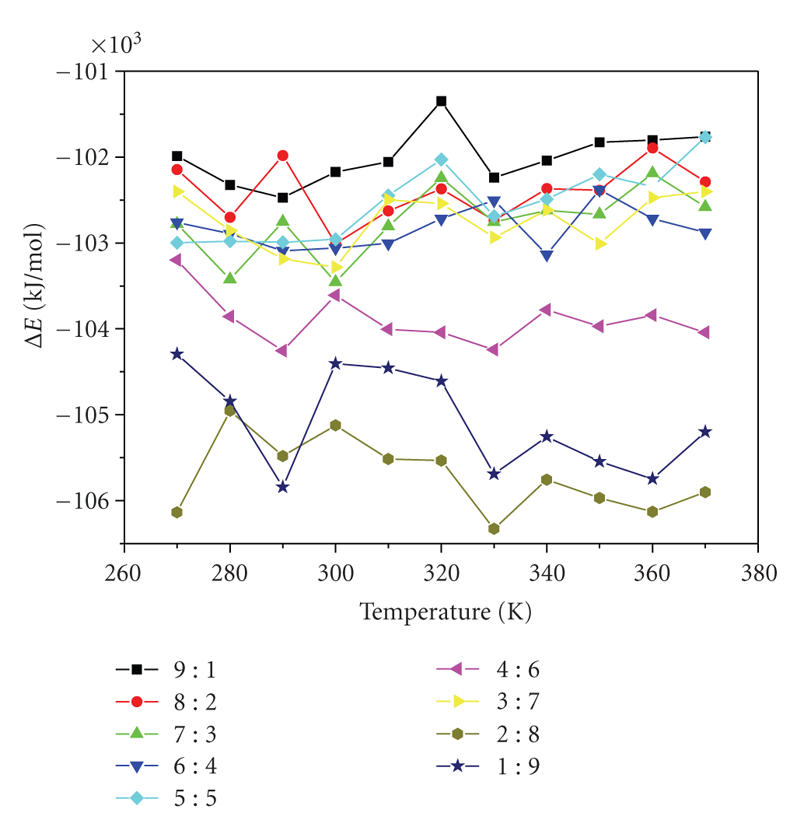
The compositive chart of Δ*E* in different crystal water proportion.

**Figure 3:**
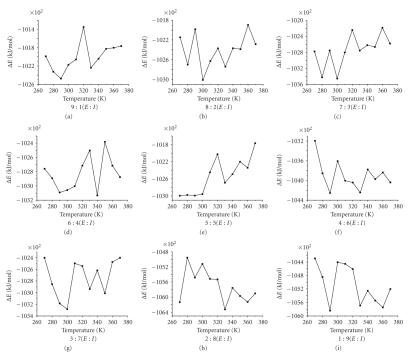
The respective chart of Δ*E* in different crystal water proportion.

The interaction energy between alpha-amylase and inhibitor covering different proportion of crystal water was calculated according to (1). The relation of the interaction energy with temperature was presented in Figure 2 (the compositive chart of Δ*E* in different crystal water proportion) and Figure 3 (the respective chart of Δ*E* in different crystal water proportion).Then, we would discuss them, respectively.

From Figure 2, it could be seen that the interaction energy between alpha-amylase and its inhibitor covering different proportion of crystal water was all negative. This information showed that it was combined and reacted between alpha-amylase and its inhibitor from 270 K to 370 K. The interaction energy was on the nadir at 330 K, when the different distributed proportion of crystal water was 2 : 8. In this condition, the reaction was the easiest between alpha-amylase and its inhibitor. However, the interaction energy was on the peak at 320 K, when the different distributed proportion of crystal water was 9 : 1. In this condition, the reaction was the hardest between alpha-amylase and its inhibitor.

From Figure 3, the optimal temperature of the interaction between alpha-amylase and its inhibitor was changed by the distributed proportion of crystal water. In the correlative study, it was reported that the optimal temperature of wheat monomeric and dimeric protein inhibitors was from 273 K to 318 K [25, 26].

The absolute value of the interaction energy was the greatest at 300 K when the distributed proportion of crystal water was 9 : 1, 8 : 2, 7 : 3, 5 : 5, 4 : 6, 3 : 7, and 1 : 9. The results showed the optimal temperature via molecular dynamics simulation which was agreed with the results of experimentation. And the binding of alpha-amylase average temperature was 289 K.The interaction energy between alpha-amylase and its inhibitor was on the nadir at 290 K in figure when the distributed proportion of crystal water was 9 : 1, 5 : 5, 4 : 6, and 1 : 9. In the case of 1 : 9, the optimal temperature may be related to some QSAR properties.

The interaction energy between alpha-amylase and its inhibitor was on the nadir at 340 K, 330 K when the distributed proportion of crystal water was, respectively, 6 : 4 and 2 : 8. These results were disagreeing with the experimental results that may be caused by the distributed proportion of crystal water and others causation, which had studied as follow.

### 3.4. Simulate optimal temperature in the case of the different distributed proportion of crystal water is 12.4%

For the sake of an accurate result, we must treat jointly the experimental results related to some QSAR properties. In the case of 1 : 9 (about 11.1%), the interaction energy between alpha-amylase and its inhibitor was the greatest in all figures above, and this proportion of crystal water was close to the data of polarizability (12.4%) in the QSAR properties. This indicated that polarizability of the QSAR properties possibly had higher influence to the interaction. We want to validate below that polarizability affected reactive temperature condition of interaction between alpha-amylase and its inhibitor.

**Figure 4:**
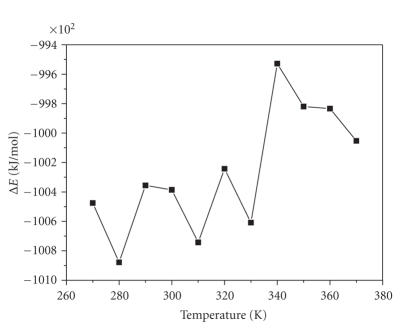
The chart of Δ*E* in polarizability crystal water proportion.

We calculated the interaction energy between alpha-amylase and its inhibitor covering (87.6 : 12.4) proportion of crystal water according to (1). The relation of the interaction energy with temperature was presented in Figure 4. From Figure 4, the optimal temperature was 280 K which was from 273 K to 318 K. However, this result was a little different to 289 K (290 k) which was average temperature in the correlative report. The proportion of Surface Area (Grid), Volume, Refractivity, and Mass effect in the interaction between alpha-amylase and its inhibitor will be studied in future.

## Figures and Tables

**Table 1:** The QSAR properties of inhibitor and alpha-amylase.

		Species	
QSAR	Inhibitor	Alpha-amylase	Inhibitor/alpha-amylase (%)
Partial charges	0.00	0.00	—
Surface area[approx.]	5220.84	over	—
Surface area[grid]	8104.71	50393.60	16.1
Volume	6860.60	43349.11	15.8
Hydration energy	2214.75	over	—
Log P	−1.71	−1218.30	0.1
Refractivity	871.39	6694.71	13.0
Polarizability	351.61	2826.71	12.4
Mass	3661.59	51193.14	7.2
